# A didactic illustration of writing skill growth through a longitudinal diagnostic classification model

**DOI:** 10.3389/fpsyg.2024.1521808

**Published:** 2025-01-15

**Authors:** Hamdollah Ravand, Farshad Effatpanah, Olga Kunina-Habenicht, Matthew J. Madison

**Affiliations:** ^1^English Department, Vali-e-Asr University of Rafsanjan, Rafsanjan, Iran; ^2^Research Unit of Psychological Assessment, Faculty of Rehabilitation Sciences, TU Dortmund University, Dortmund, Germany; ^3^Department of Educational Psychology, University of Georgia, Athens, GA, United States

**Keywords:** feedback, diagnostic classification models, growth modeling, TDCM, L2 writing

## Abstract

**Introduction:**

Diagnostic classification models (DCMs) have received increasing attention in cross-sectional studies. However, L2 learning studies, tracking skill development over time, require models suited for longitudinal analyses. Growth DCMs offer a promising framework for such analyses.

**Method:**

This study utilizes writing data from two learner groups: one receiving peer feedback (*n* = 100) and the other receiving no feedback (*n* = 100), assessed at three time points. It demonstrates the application of longitudinal DCM via the TDCM package to analyze growth trajectories in four writing subskills: Content, Organization, Grammar, and Vocabulary. The primary focus is on showcasing the package, but substantive findings can also be helpful.

**Results:**

The multi-group analysis revealed similar V-shaped growth trajectories for Grammar and Vocabulary, along with consistent inverted V-shaped patterns for Organization and Content in both groups.

**Discussion:**

The results showed minor differences between the two groups, potentially indicating the limited impact of peer feedback on L2 writing development. This could be attributed to the social dynamics between peers.

## 1 Introduction

In the last few years, there has been a growing research interest in diagnostic classification models (DCMs), usually referred to as cognitive diagnostic models (CDMs; e.g., Rupp et al., 2010), among researchers and practitioners in the field of educational assessment, in general, and language assessment and testing, in particular. With the primary goal of assessment being the identification and improvement of learning outcomes (Stiggins, 2002), DCMs serve as psychometric frameworks facilitating formative assessment by offering detailed diagnostic feedback on students’ strengths and weaknesses (DiBello et al., 2007).

A large number of studies have been conducted to utilize DCMs across various second/foreign language (L2) skills to both uncover the *processes, subskills, or* attributes essential for successfully accomplishing tasks/items and diagnose language ability of students (Buck and Tatsuoka, 1998; Chen and Chen, 2016; Lee and Sawaki, 2009; Sawaki et al., 2009; von Davier, 2008; Yi, 2017). While these prior investigations have yielded valuable insights into the effectiveness of DCMs in language testing and assessment, they primarily concentrated on receptive skills (i.e., reading and listening). A handful of studies have also used dichotomous (Effatpanah et al., 2019; He et al., 2021; Kim, 2010; Ravand et al., 2024; Xie, 2016) and polytomous (e.g., Shi et al., 2024) DCMs to diagnose L2 writing ability of students. Although the studies could show the feasibility of using DCMs to diagnose L2 students’ language skills, the majority of these applications have been confined to one-off implementations in cross-sectional studies. Several researchers have recently developed longitudinal DCMs (e.g., Chen et al., 2018; Lin et al., 2020; Madison and Bradshaw, 2018; Pan et al., 2020; Wen et al., 2020; Zhan, 2020; Zhan et al., 2019; Zhang and Wang, 2018) to measure changes in attribute mastery status over a period of time. To the best of the authors’ knowledge, only few studies (e.g., Chen et al., 2018; Lin et al., 2020; Zhang and Wang, 2018) have already used longitudinal DCMs in educational assessment contexts. Too little attention has been devoted to the application of longitudinal DCMs in assessing language components and skills.

Against this background, the present study aims to illustrate the use of a growth diagnostic classification model (Madison and Bradshaw, 2018), as implemented in the TDCM package (Madison et al., 2024), to track changes in language learners’ writing data from two learner groups (i.e., one receiving peer feedback and the other receiving no feedback) assessed at three time points. This model operates at a fine-grained level of subskills, offering detailed insights that help tailor instruction to the evolving needs of language learners over time. Specifically, the growth DCM provides a robust framework for capturing developmental changes in L2 writing skills, enabling researchers to examine how different instructional interventions affect subskill mastery in experimental settings. While the primary focus of this study is to demonstrate the application of the TDCM package, a secondary aim is to explore the substantive insights derived from the results, particularly regarding the impact of peer feedback on L2 learners’ writing performance.

Building on this foundation, growth DCMs (e.g., Chen et al., 2018; Kaya and Leite, 2017; Li et al., 2016; Madison and Bradshaw, 2018; Wang et al., 2018; Wen et al., 2020; Zhang and Wang, 2018) combine the analytical power of longitudinal models with the diagnostic precision of DCMs. They capture individual trajectories of change over time, offering a nuanced understanding of developmental processes in second language acquisition. By delineating these changes within individuals, growth DCMs reveal patterns of growth, stability, or decline that might otherwise remain obscured in cross-sectional analyses. Furthermore, multiple-group growth models enable simultaneous examination of inter-individual differences (e.g., between learner groups) and intra-individual changes (e.g., within learners over time), providing critical insights into both the variability of developmental trajectories and the contextual factors shaping them. By delivering detailed diagnostic feedback on learners’ mastery or non-mastery of subskills across different time points, growth DCMs are particularly suited for evaluating the impact of instructional interventions, making them a valuable tool in experimental and quasi-experimental educational research.

## 2 Background

### 2.1 Peer feedback

Peer feedback has been shown to facilitate second language acquisition from social, affective, and linguistic perspectives. From a social standpoint, peer feedback aligns with Vygotsky’s Zone of Proximal Development (ZPD), creating a collaborative environment where students support each other’s learning (Mendonça and Johnson, 1994; Tsui and Ng, 2000). This social interaction enhances awareness of audience considerations and encourages an audience-centered approach to writing, motivating students to invest more effort and take ownership of their work. In turn, the peer feedback process acts as a scaffold, enabling students to perform tasks they may not be able to achieve independently, thus advancing their learning within the ZPD framework.

Affectively, peer feedback helps reduce defensive reactions to critique, leading to more positive attitudes toward writing. This is consistent with Krashen’s Affective Filter Hypothesis, which suggests that lowering anxiety levels can facilitate better language acquisition (Higgins et al., 2002; Gielen et al., 2010; Min, 2006). By creating a supportive atmosphere, peer feedback encourages greater participation and acceptance of constructive criticism, helping students feel more comfortable and motivated to engage in the writing process.

Linguistically, peer feedback serves as a catalyst for second language acquisition and oral fluency development by exposing students to a variety of language structures and vocabulary. This supports Long’s Interaction Hypothesis, which suggests that language proficiency improves through meaningful interaction and feedback (Yu and Lee, 2016). Engaging in peer feedback activities allows students to practice and refine their language skills in context, reinforcing learning and retention of new language concepts.

### 2.2 Diagnostic classification models (DCMs)

DCMs are psychometric models primarily designed to evaluate students’ levels of mastery or non-mastery across various attributes (DiBello et al., 2007). In contrast to conventional psychometric models, such as classical test theory (CTT) or item response theory (IRT), which assume a true score or latent trait to position students along a continuum based on their assessment performance, DCMs generate *skill profiles* or *profile scores.* These scores are expressed dichotomously, indicating whether a student has *mastered* or *not mastered* each specific skill or attribute being assessed. This detailed breakdown of strengths and weaknesses enables educators to offer more targeted instruction and personalized remedial strategies based on the individual needs of each student (Kunina-Habenicht et al., 2009).

A wide array of DCMs has been formulated, each grounded in distinct assumptions or theories about the way cognitive processes, (sub)skills, or attributes impact students’ responses during assessment. The deterministic inputs, noisy, “and” gate (DINA) model is an example of non-compensatory DCMs assuming that one must master all the required attributes to correctly answer a given item. However, in compensatory models, mastery of any of the attributes can compensate for the non-mastery of the other attributes. For example, the deterministic inputs, noisy, “or” gate (DINO; Templin and Henson, 2006) model, as a prime example of compensatory DCMs, assumes that the mastery of at least one of the required attributes is required for correctly answering an item. With additive DCMs, mastery of each attribute leads to increase in success probability regardless of mastery or non-mastery of the other attributes. In fact, each attribute contributes to the probability of a correct response in and of itself; if it has been mastered, it would increase the probability of the correct response; if not, it does not nullify the effect of the other required attributes. Examples of additive DCMs are the linear logistic model (LLM; Maris, 1999), the reduced reparametrized unified model (RRUM), and the additive CDM (*A*-CDM; de la Torre, 2011).

In addition to these specific DCMs, several general DCMs have been developed that allow all three types of relationships within the same test, such as the general diagnostic model (GDM; von Davier, 2008), the log-linear cognitive diagnosis model (LCDM; Henson et al., 2009), and the generalized DINA (G-DINA; de la Torre, 2011) model. de la Torre (2011) demonstrated that when appropriate constraints are imposed to the parametrization of general DCMs, several specific DCMs as special cases of the general models can be obtained. For instance, the DINA and DINO models can be obtained from the G-DINA when the main effects and the interaction effects are set to zero. The additive DCMs (i.e., the *A*-CDM, RRUM, and LLM) can be derived by setting all the interaction effects to zero in identity, log, and logit link G-DINA model, respectively.

### 2.3 Longitudinal diagnostic classification models

An important topic in educational research and assessment revolves around measuring the constant change of students’ knowledge and skills over time (Fischer, 1995a), often influenced by various instructional interventions. Understanding the quantitative aspect of students’ learning trajectories or cognitive development is crucial for researchers and educators. Traditional approaches, like the CTT, utilize gain scores, which measure the difference in total test scores across different testing occasions (e.g., pre-and post-tests), to provide measures of students’ growth (Williams and Zimmerman, 1996). However, despite its simplicity, gain-score-based methods exhibit inadequate psychometric properties and reliability (Linn and Slinde, 1977). To address this, researchers have turned to psychometric models that treat students’ knowledge and skills as latent constructs. Within the IRT framework, numerous longitudinal models have been proposed to assess growth in both individual and group abilities along a proficiency continuum (Andersen, 1985; Andrade and Tavares, 2005; Embretson, 1991; Fischer, 1989, 1995b; Huang, 2015). Nonetheless, these models are limited in their ability to capture growth in categorical latent trait variables and often fall short in providing detailed insights into students’ strengths and weaknesses across various attributes over time.

Over the past few years, longitudinal learning diagnosis has received a great deal of attention due to the importance of assessing changes in attribute mastery status and profiles over time. A variety of longitudinal DCMs have been developed to capture these changes which can be categorized into two main groups. The first group includes models based on latent transition analysis (LTA; Collins and Wugalter, 1992) that are utilized to estimate the probabilities of transitions in attribute mastery across time (e.g., Chen et al., 2018; Kaya and Leite, 2017; Li et al., 2016; Madison and Bradshaw, 2018; Wang et al., 2018; Wen et al., 2020; Zhang and Wang, 2018). The second group consists of models based on higher-order latent structural analysis (de la Torre and Douglas, 2004), which track changes in higher-order latent traits over time to deduce shifts in attributes (e.g., Huang, 2017; Lee, 2017; Lin et al., 2020; Pan et al., 2020; Zhan, 2020; Zhan et al., 2019). Recently, Madison and Bradshaw (2018) developed the Transition Diagnostic Classification Model (TDCM) which is an integration of LTA with general DCMs (e.g., the G-DINA and LCDM), to offer a method for analyzing growth in a general DCM framework. Since G-DINA and LCDM are considered general DCMs, various other DCMs can be encompassed within TDCM.

LTA is considered a specialized form of the latent or hidden Markov model (HMM; Baum and Petrie, 1966) and serves as an extension of the latent class model within longitudinal studies. Within the framework of LTA, the membership of individuals in classes at each time point remains latent, yet is inferred from a set of observable item responses. The measurement model employed in LTA, which characterizes the probabilities of item responses at each time point, is a latent class model. Moreover, transitions between classes over time are delineated through latent class transition probabilities, offering sequential progressions across time points (Madison and Bradshaw, 2018). In conventional latent class analysis, the determination of the number of latent classes typically involves an exploratory approach. Multiple models are tested, each with varying numbers of latent classes, and the model demonstrating the best fit, often characterized by parsimony, is selected for interpretation (Collins and Lanza, 2010). This methodology parallels the approach taken in LTA, where the number of latent classes at each time point is established through comparisons of LTAs with differing numbers of latent classes across time points.

As a saturated and general DCM, the LCDM (Henson et al., 2009) offers a flexible framework to empirically examine associations between items and attributes through different parameter specifications. The model parametrizes the probability of giving a correct answer to a given item as a function of the attributes measured by the item, student attribute mastery, and the item parameters. The item response function of the LCDM for an item measuring two attributes is as follows:

(1) $$P\left(X_{jc}=1|\alpha_{c}\right)=\frac{\exp\left(\lambda_{j,0}+\lambda_{j,1,\left(2\right)}\left(a_{c2}\right)+\lambda_{j,1,\left(3\right)}\left(a_{c3}\right)+\lambda_{j,2,\left(2,3\right)}\;\left(a_{c2}.a_{c3}\right)\right)}{1+\exp(\lambda_{j,0}+\lambda_{j,1,\left(2\right)}\left(a_{c2}\right)+\lambda_{j,1,\left(3\right)}\left(a_{c3}\right)+\lambda_{j,2,\left(2,3\right)}\;\left(a_{c2}.a_{c3}\right)}$$

In Equation 1, $X_{jc}$ denotes the random variable to item j by a student with the specific attribute profile $\alpha_{c}$ (c refers to the index of the specific attribute profile); $\lambda_{j,0}$ is the intercept representing the log-odds of a correct response for the reference group—students who have not mastered Attribute 2 or Attribute 3; $\lambda_{j,1,\left(2\right)}$ and $\lambda_{j,1,\left(3\right)}$ are the main effects associated with Attributes 2 and 3, respectively. These parameters indicate the increase in log-odds of a correct response for students who have mastered Attribute 2 or Attribute 3 independently; lastly, $\lambda_{j,2,\left(2,3\right)}$ indicates the interaction term capturing the additional change in log-odds for students who have mastered both Attribute 2 and Attribute 3. The magnitude of these parameters quantify how mastery of specific attributes influences the probability of a correct response.

Just as a DCM which is a confirmatory latent class model with predetermined latent classes, representing attribute profiles, the TDCM is a confirmatory LTA with the latent classes at each time point predefined as attribute profiles (Madison and Bradshaw, 2018). Consider a student v answering to J items across T testing occasions. The probability of success for student v is expressed as:

(2) $$P\left(X_{j}=1\right)=\overset{C}{\underset{c_{1}=1}{\sum }}\overset{C}{\underset{c_{2}=1}{\sum }}\ldots \overset{C}{\underset{c_{T}=1}{\sum }}m_{c_{1}}\tau_{c_{2}|c_{1}}\tau_{c_{3}|c_{2}}\ldots \tau_{c_{T}|c_{T-1}}\;\overset{T}{\underset{t=1}{\prod }}\overset{J}{\underset{j=1}{\prod }}\pi_{jc_{t}}^{x_{vjt}}\;{\left(1-\pi_{jct}\right)}^{1-x_{vjt}}$$

In Equation 2, $m_{c_{1}}$ signifies the probability of membership in Attribute Profile c at Time Point 1. As defined by Madison and Bradshaw (2018), each sum encompasses all C attribute profiles for each testing occasion. The first product term spans across the T testing occasions, while the second product term spans across the J items. $x_{vjt}$ is Student v ’s response to Item j at Testing Occasion t ; the $\pi_{jct}$ ’s denote the item response probabilities; and each $\tau_{c_{t}|c_{t-1}}$ indicates the probability of transitioning between different attribute mastery statuses between Testing Occasion t−1 to Testing Occasion t .

While the usefulness of the longitudinal DCMs has been assessed in analyzing learning diagnosis data, the majority of existing applications in the literature have been add-ons to simulation studies for model development and refinement. DCMs have been utilized in one-off studies to demonstrate their applicability to language skills such as L2 writing (e.g., Kim, 2010; Xie, 2016), explore the relationship between subskills of L2 writing (e.g., Ravand et al., 2024) and examine writing proficiency of a group of learners of English as a foreign language at the fine-grained level of subskill (e.g., Effatpanah et al., 2019; He et al., 2021; Shi et al., 2024). Notably, there has been a dearth of empirical studies applying longitudinal DCMs in educational settings (e.g., Lin et al., 2020), and to the best knowledge of the authors, no study has ever employed DCMs to measure the development of language skills, especially L2 writing ability, over time.

## 3 The present study

The aim of this study is to demonstrate the feasibility of using the TDCM (Madison and Bradshaw, 2018) to track changes in attribute mastery status over time utilizing the TDCM package (Madison et al., 2024) in R (R Core Team, 2024). To achieve this, our analysis focused on examining potential differences between the feedback and no-feedback groups in terms of their growth trajectories across four attributes and multiple time points. Additionally, as a secondary objective, the study explores the substantive implications of the effect of peer feedback on L2 learners’ writing performance.

## 4 Method

### 4.1 Data

For this demonstration, a segment of data from an ongoing larger study exploring the impact of three types of feedback on the development of subskills in L2 writing was utilized. Originally, the study comprised three groups: one receiving peer feedback, another receiving teacher-mediated peer feedback, and a third receiving no feedback. For the purpose of the present study, some participants from the no-feedback group (NFG) and peer-feedback group (PFG) were chosen. Since the original feedback study, which began in the fall semester of 2022, was still ongoing during the writing of this paper, this study included only 200 participants, divided into two groups of 100 each. Participants consisted of students majoring in English Language Teaching, English Literature, and Translation Studies at three state universities in Iran. Since the participants were in the second year of their Bachelor’s studies and all had been admitted into their respective universities through the university entrance examination which requires intermediate to upper intermediate knowledge of vocabulary, grammar, and reading comprehension, we assumed that they were intermediate to upper intermediate English learners. It is worth noting that although we used equal group sizes due to data availability limitations, the TDCM package is fully capable of handling uneven group sizes as well.

### 4.2 Procedure and Q-matrix development

Starting the second session of the respective semesters, students wrote six paragraphs of at least 200 words over six consecutive weeks during regular paragraph writing courses. In the PFG, participants provided comments on anonymously submitted paragraphs from their peers, which were then revised accordingly. These peer comments and the subsequent revisions were submitted before the following session. Conversely, participants in the NFG solely received instructions on paragraph writing techniques and completed exercises aimed at enhancing their paragraph writing skills, without receiving any feedback.

At intervals of 3 weeks, 5 weeks, and 7 weeks into the study, the writing abilities of both groups were evaluated. They were tasked with composing paragraphs of 200–250 words on three distinct yet related prompts. The quality of these paragraphs was assessed using a descriptive-based diagnostic checklist developed by Kim (2010). This checklist comprised 35 descriptors evaluating five subskills: content fulfillment (CON), organizational effectiveness (ORG), grammatical knowledge (GRM), vocabulary use (VOC), and mechanics (MCH) as shown in Table 1. Each descriptor is accompanied by *yes* or *no* response option. If a rater assumes that the writer *generally meets* the criterion explained in any given descriptor, a *yes* is suggested; otherwise, the recommendation is a *no*. When a rater’s comprehension of the text is not disrupted by violations of the skill under assessment, the writer is said to generally meet the criterion in the descriptor.

**Table 1:** The Five Writing Subskills Definition.

Writing Subskills	Description
Content Fulfillment (CON)	Content fulfillment is the extent to which a writer can address a prompt by demonstrating unity and appropriacy of supporting sentences, ideas, information, and examples.
Organizational Effectiveness (ORG)	Organizational effectiveness is the extent to which a writer can generate and organize ideas cohesively and coherently within and between sentences and paragraphs.
Grammatical Knowledge and Mechanics (GRM)	Grammatical knowledge is the degree to which a writer can build complex sentences and use variety of grammatical structures accurately; Mechanics is the degree to which a writer can demonstrate the correct conventions and styles of English writing such as margins, indentation, punctuation, spelling and capitalization.
Vocabulary Use (VOC)	Vocabulary use is the degree to which a writer can use variety of accurate and appropriate vocabulary items and collocations, and demonstrate the knowledge of word form.

The relationships between the checklist descriptors and the subskills were outlined in the Q-matrix provided in Table 2. A Q-matrix is a tabular representation where rows correspond to test items and columns correspond to attributes or subskills measured by those items. Entries of “0” indicate that the item does not measure the attribute, while “1” indicates that it does.

**Table 2:** Final Q-matrix.

Item	GRM	ORG	CON	VOC
1	0	1	0	1
2	1	0	1	0
3	1	0	1	0
4	0	1	1	0
5	0	1	1	0
6	0	0	1	0
7	0	1	1	0
8	0	0	1	0
9	0	1	0	0
10	0	1	0	0
11	0	1	0	0
12	0	1	1	0
13	0	1	1	0
14	1	1	0	1
15	1	0	0	0
16	1	0	0	0
17	1	0	0	0
18	1	0	0	0
19	1	0	0	0
20	1	0	0	0
21	1	0	0	0
22	1	0	0	0
23	1	0	0	0
24	1	0	0	0
25	1	0	0	0
26	0	0	0	1
27	0	0	0	1
28	0	0	0	1
29	1	0	0	1
30	1	0	0	0
31	1	0	0	0
32	1	0	0	0
33	1	0	0	0
34	1	1	0	0
35	0	0	0	1

GRM, Grammar and Mechanics; ORG, Organization; CON, Content; VOC, Vocabulary.

We conducted the model analysis utilizing the full Q-matrix, which included five attributes. Unfortunately, the model failed to converge, prompting an investigation into potential contributing factors. The relatively small sample size and the number of time points emerged as primary suspects in this scenario. Due to the unavailability of additional data at the time, augmenting the sample size was not a viable option. Furthermore, given the pioneering nature of our study in illustrating the application of the TDCM and the rarity of research demonstrating the use of growth DCMs, coupled with the observation that most longitudinal studies typically encompass more than two time points, we opted to proceed with three time points.

In an attempt to address the convergence issue, we experimented with merging the Mechanics attribute with Grammar, thereby reducing the number of attributes in the Q-matrix to four, as depicted in Table 2. This alteration proved effective in achieving model convergence. The decision to proceed with this merging was based on the expert judgment of the authors, all of whom have over 10 years of experience in researching and applying DCMs. This decision was further supported by literature indicating that as the number of attributes increases in DCMs, a larger sample size is required to ensure stable and reliable estimation of model parameters. Two raters, each with at least 5 years of experience in teaching and assessing writing in higher education, evaluated the paragraphs. Prior to rating, they underwent training. Inter-rater reliability was calculated by having the two raters assess the same 20 papers using the checklist. The Cohen’s Kappa index indicated that the raters agreed 79% of the time. It should be noted that details of Q-matrix validation are omitted from this paper due to space constraints and the abundance of literature (e.g., Ravand, 2016; Ravand and Robitzsch, 2018) illustrating Q-matrix specification and empirical validation. Consequently, the primary focus of this paper is on demonstrating the application of the under-illustrated growth DCMs.

Since the same checklist was employed across all three time points, the Q-matrix remained consistent throughout the study. It is important to note that the TDCM package has the capability to handle data from various tests administered at different time points, each measuring different attributes. In DCM terms, the TDCM package can accommodate different Q-matrices at different time points. While this study measures the same attributes at all three time points, the annotated R code in Appendix illustrates the specification of models with different Q-matrices at varying time points.

It should be noted that in the present study, we conducted two rounds of analysis. In the first round, the entire available dataset was analyzed without considering the grouping of subjects. In the second round, using the growth DCM, we examined the development of subskills between the peer-feedback and no-feedback groups over time. However, due to the similarity of the analyses and in the interest of space, we have presented only the multigroup results in this paper.

## 5 Results

### 5.1 Assessment of measurement invariance

Before examining growth trajectories of the attributes across the groups and time points, the best-fitting model was first selected. In the present study, four models were estimated and compared. The models were as follows:

Multigroup 1: This model assumes complete invariance of item parameters, meaning that the same item parameters are applied across both groups and time points. By enforcing strict invariance, it serves as a baseline for evaluating changes over time or between groups.
Multigroup 2: This model assumes invariance across time points but allows for differences between groups. Here, the item parameters are held constant over time, but not between the groups, enabling an examination of how groups differ in their response patterns while controlling for temporal stability.
Multigroup 3: this model assumes invariance across groups but allows item parameters to vary across time points. This approach is useful for exploring how individual changes in performance over time can be modeled while assuming the groups have similar item parameters.
Multigroup 4: The final model assumes no invariance across either groups or time points, allowing item parameters to vary freely. This more flexible model is crucial for identifying potential differences in item functioning across both dimensions, providing insights into whether the groups or the time points exhibit distinct patterns in their responses.

As Table 3 shows, the log likelihood values and in turn the deviances for all the models were smaller than those of the Multigroup1, and the *p*-values for the chi square difference tests showed that the differences are significant, hence the assumption of invariance of item parameters across both groups and time points did not hold. From among the other models, Multigroup 3 which had the smallest AIC and BIC values was chosen for the rest of multigroup analyses.

**Table 3:** Comparing models with varying assumptions.

Model	Loglike	Deviance	Npars	AIC	BIC	Chisq	df	*p*-value
mg1	−22784.2	45568.43	220	46008.43	46975.75	149.12	62	0
mg2	−22709.6	45419.31	282	45983.31	47223.24	NA	NA	NA
mg3	−22302.4	44604.94	344	45292.94	46805.48	NA	NA	NA
mg4	−22155.7	44311.54	530	45371.54	47701.91	NA	NA	NA

Npars, Number of Parameters; df, degrees of freedom; mg, Multigroup.

Regarding the absolute fit indices, the following fit indices were consulted: Max(X2), abs(fcor), RMSEA, and SRMSR. The test-level Max(X2) (Chen and Thissen, 1997) is derived from pair-wise X2 values, averaged across all item pairs. It reflects the average difference between model-predicted and observed response frequencies. High Max(X2) values suggest the presence of unmodeled residual local dependencies between items. A non-significant MX2 value indicates a well-fitting model. For SRMSR and RMSEA, values < 0.05 have been suggested as showing substantively negligible amount of misfit (Maydeu-Olivares, 2013). In addition to the above-mentioned indices, a residual-based statistic, transformed correlation (abs(fcor)), was also examined. Abs (fcor) is the residual between the observed and predicted Fisher-transformed correlation of item pairs. According to when the model fits the data, the value of this residual-based statistic should be close to zero for all items. Values not significantly different from zero, as indicated by Bonferroni adjusted *p*-values > 0.05, indicate a well-fitting model.

As shown in Table 4, the indices collectively suggested that Multigroup 3 fits the data best. The non-significant values for max(X2) and abs(fcor), along with an SRMSR value of 0.04 (below the 0.05 threshold) and an RMSEA value just above 0.05, supported its superior fit compared to the other models.

**Table 4:** Absolute fit indices.

Model	Max(X2)	*p*-value	Abs(fcor)	*p*-value	RMSEA	RSMSR
mg1	57.27	0.00	0.23	0.00	0.16	0.12
mg2	35.02	0.00	0.29	0.00	0.54	0.91
mg3	11.67	1.00	0.14	0.53	0.06	0.04
mg4	18.06	0.04	0.20	0.00	0.16	0.12

mg, Multigroup.

### 5.2 Item parameters

The TDCM generates the log odds ratios for each item parameter. Since Multigroup 3 was selected as the best model, the assumption is that item parameters vary across the three time points while remaining invariant across groups. Accordingly, the TDCM package produces one set of item parameters for each time point. If Multigroup 4, which assumes invariance neither across groups nor time points, had been selected, the package would have generated six sets of item parameters, one for each combination of group and time point. In the interest of space, the item parameters for the first three items across the three time points have been reproduced in Table 5. For the ease of interpretation, the log odds ratios have been converted to probabilities using the following formula: $\frac{1}{1+e^{-\left(\log\mathrm{odds}\right)}}$ .

**Table 5:** Item parameters.

Time Points	Items	λ0	λ1,1	λ1,2	λ1,3	λ1,4	λ2,12	λ2,13	λ2,24
T1	Item 1	0.34	–	0.45	–	0.22	–	–	0.53
	Item 2	0.51	0.34	–	0.32	–	–	0.32	–
	Item 3	0.36	0.46	–	0.52	–	–	0.46	–
T2	Item 1	0.26	–	0.16	–	0.73	–	–	0.60
	Item 2	0.74	0.40	–	0.22	–	–	0.21	–
	Item 3	0.39	0.36	–	0.52	–	–	0.46	–
T3	Item 1	0.44	–	0.31	–	0.48	–	–	0.37
	Item 2	0.46	0.36	–	0.11	–	–	0.34	–
	Item 3	0.30	0.42	–	0.76	–	–	0.41	–

In Table 5, λ0 is the base-rate probability which represents the probability of getting the given item right when none of the required attributes for the item has been mastered, and the correct answer comes from guessing. Additionally, λ1,1; λ1,2; λ1,3; and λ1,4 denote the increase in the probability of getting any given item right when Attributes 1, 2, 3, and 4 (i.e., Grammar, Organization, Content, and Vocabulary, respectively) have been mastered, respectively, compared to the base rate probability. Furthermore, λ2,12; λ2,13, and λ2,24 indicate the increase in the probability of getting the given item right when both Attributes of Grammar and Organization, Grammar and Content, and Organization and Vocabulary have been mastered, compared to the base-rate probability.

As depicted in Table 5, the parameters for the three sample items varied across all the time points. To illustrate, let us analyze the parameters for Item 1, which necessitates Organization and Vocabulary, across the initial and subsequent time points. At Time 1, individuals who have not mastered the two required attributes have a 34% probability of answering the item correctly (i.e., item intercept). For those who have only mastered Organization, the likelihood of answering correctly increases by 45% compared to those who have not mastered any attribute. Similarly, individuals who have only mastered Vocabulary exhibit a 22% higher probability of answering correctly compared to those who have not mastered any attribute. Moreover, for individuals who have mastered both Organization and Vocabulary, there is 53% increase in the probability of answering the item correctly compared to those who have not mastered any attributes. The corresponding probabilities for the same item at Time 2 are 26%, 16%, 73%, and 60%, while they are 44%, 31%, 48%, and 37% for Time 3. A comparison of the parameters for Item 1 across time revealed that the guessing parameter is the smallest at Time 2. Additionally, at Time 1, Organization better discriminates between those who have mastered the attribute and those who have not, while at Time 2 and 3, Vocabulary exhibits superior discrimination between its masters and non-masters.

### 5.3 Transition probabilities across groups

In the multigroup analysis, the TDCM package yields average mastery probabilities across the different time points. Table 6 shows the growth of the attributes across the two groups and the three time points.

**Table 6:** Growth across the groups and time points.

NFG				PFG			NFG		PFG		NFG	PFG
	T1	T2	T3	T1	T2	T3	T1-T2	T1-T3	T1-T2	T1-T3	Odds Ratio	Odds Ratio
GRM	0.67	0.41	0.79	0.67	0.44	0.79	−0.27	0.12	−0.23	0.12	1.18	1.18
ORG	0.37	0.61	0.48	0.41	0.77	0.49	0.24	0.11	0.36	0.08	1.30	1.20
CON	0.62	0.83	0.52	0.72	0.87	0.53	0.21	−0.10	0.15	−0.19	0.84	0.74
VOC	0.46	0.54	0.70	0.58	0.34	0.63	0.08	0.25	−0.24	0.06	1.52	1.09

NFG, no feedback group; PFG, peer feedback group; GRM, Grammar and Mechanics; ORG, Organization; CON, Content; VOC, Vocabulary.

Across both NFG and PFG, the mastery trajectories of the attributes revealed dynamic patterns across the three time points. Initially, both groups started with varying levels of mastery probabilities. For example, for Grammar, both groups started with a mastery probability of 67% at Time 1, but this decreased to 41% for NFG and 44% for PFG at Time 2 before increasing to 79% for both groups at Time 3. Similarly, for Content, both groups started with relatively high mastery probabilities at Time 1 (62% for NFG and 72% for PFG), increased substantially at Time 2 (83% for NFG and 87% for PFG), and decreased at Time 3 (52% for NFG and 53% for PFG). However, Vocabulary showed different patterns, with NFG demonstrating a consistent increase in mastery probability from 46% at Time 1 to 70% at Time 3, while PFG exhibited fluctuations, decreasing to 34% at Time 2 before increasing to 63% at Time 3. Overall, while both groups generally showed improvements in mastery probabilities over time for most attributes, the magnitude and consistency of these changes varied between the groups and attributes.

To gain deeper insights into the growth trajectories of attributes across the groups, we manually subtracted the mastery probabilities of the attributes at Time 1 from those at Time 2 and Time 3. The results of these subtractions are presented in Columns 8 to 11 of Table 6, respectively. The analysis across the four attributes revealed several trends regarding the mastery probabilities and the impact of the intervention on both NFG and PFG. For Grammar, both groups experienced a decrease in mastery probabilities from Time 1 to Time 2 (−0.27 for NFG, −0.23 for PFG), followed by an increase from Time 1 to Time 3 (0.12 for both groups). This initial decline was overcome by an overall growth from Time 1 to Time 3 (0.12 for both groups). Similarly, for Organization, both groups showed increases in mastery probabilities from Time 1 to Time 2 (0.24 for NFG, 0.36 for PFG), followed by further increases from Time 1 to Time 3 (0.11 for NFG, 0.08 for PFG), suggesting a consistent improvement resulting from the intervention. However, for Content, NFG demonstrated a slight overall decrease from Time 1 to Time 3 (-0.10), while PFG showed a more significant decrease over the same period (−0.19), indicating reduced effectiveness or potential regression despite the intervention. Vocabulary, on the other hand, showed a small increase in mastery probabilities for NFG (0.08) but a notable decrease for PFG (−0.24) from Time 1 to Time 2, followed by further increases from Time 1 to Time 3 (0.25 for NFG, 0.06 for PFG), suggesting a positive impact of the intervention. In summary, the overall trends indicated growth or improvement in mastery probabilities resulting from the intervention, with variations observed across different attributes and groups, highlighting the nuanced effectiveness of the intervention.

The TDCM package does not generate significance tests for mastery probabilities across groups, which is justified in the context of DCM where the sample sizes are relatively large and even differences as small as 0.01 can be statistically significant. In light of this and following Madison and Bradshaw (2018), we computed odds ratio effect sizes by dividing the mastery probabilities at each time point by the corresponding probabilities at the preceding time point. An odds ratio of 1.52, for instance, would indicate that the odds of mastery at posttest are 1.52 times the odds of mastery at pretest. In order not to clutter the table, we computed the odds ratios only for Time 1 to Time 3 (mastery probabilities at Time 3/mastery probabilities at Time 1). As the last two columns of Table 6 show, the effect sizes for the two groups across that attributes were very close, indicating that the use of peer feedback did not increase the chances of mastery of the attributes.

Table 7 is perhaps the most important output the TDCM generates. For each attribute, the following cells are of paramount importance: the cells at the intersection of “0” (in the rows) and “1” (in the columns) denoting transitioning from non-mastery to mastery, hence occurrence of learning and “1” and “0” signifying transitioning from mastery to non-mastery (also referred to as regression, attrition, or forgetting in this paper). As depicted in Table 7, a notable trend emerged: for all attributes except for Vocabulary and Content, the growth values for PFG during the first-time interval (Time 1 to Time 2) consistently surpassed those of NFG, while the attrition rates consistently fell below their counterparts in NFG. Conversely, during the second time interval (Time 2 to Time 3), this trend reversed: the growth values for NFG, except for Content, consistently exceeded those of PFG. Similarly, the attrition rates for all attributes in NFG, except for Grammar, were lower than those of PFG. Overall, during the first-time interval, PFG outperformed NFG with regard to both higher rates of learning and lower rates of attrition, whereas during the second time interval, NFG outpaced PFG with regard to both higher learning and lower attrition rates.

**Table 7:** Multigroup transition probabilities.

		Time 1 to Time 2								Time 2 to Time 3							
		GRM		ORG		CON		VOC		GRM		ORG		CON		VOC	
NFG		0	1	0	1	0	1	0	1	0	1	0	1	0	1	0	1
	0	0.74	**0.26**	0.36	**0.64**	0.01	**0.99**	0.48	**0.53**	0.14	**0.86**	0.98	**0.02**	0.97	0.**04**	0.05	**0.95**
	1	*0.52*	0.48	*0.43*	0.57	*0.28*	0.72	*0.64*	0. 36	*0.31*	0.69	*0.23*	0.78	*0.38*	0.62	*0.51*	0.49
PFG		0	1	0	1	0	1	0	1	0	1	0	1	0	1	0	1
	0	0.71	**0.29**	0.15	**0.85**	0.16	**0.84**	0.70	**0.30**	0.24	**0.76**	1.00	**0.00**	0.77	**0.23**	0.25	**0.75**
	1	*0.44*	0.56	*0.36*	0.64	*0.12*	0.88	*0.45*	0.55	*0.18*	0.82	*0.36*	0.64	*0.43*	0.57	*0.60*	0.40

NFG, no feedback group; PFG, peer feedback group; GRM, Grammar and Mechanics; ORG, Organization; CON, Content; VOC, Vocabulary. Bold values indicate growth, while values in italic represent attrition.

**Figure 1:**
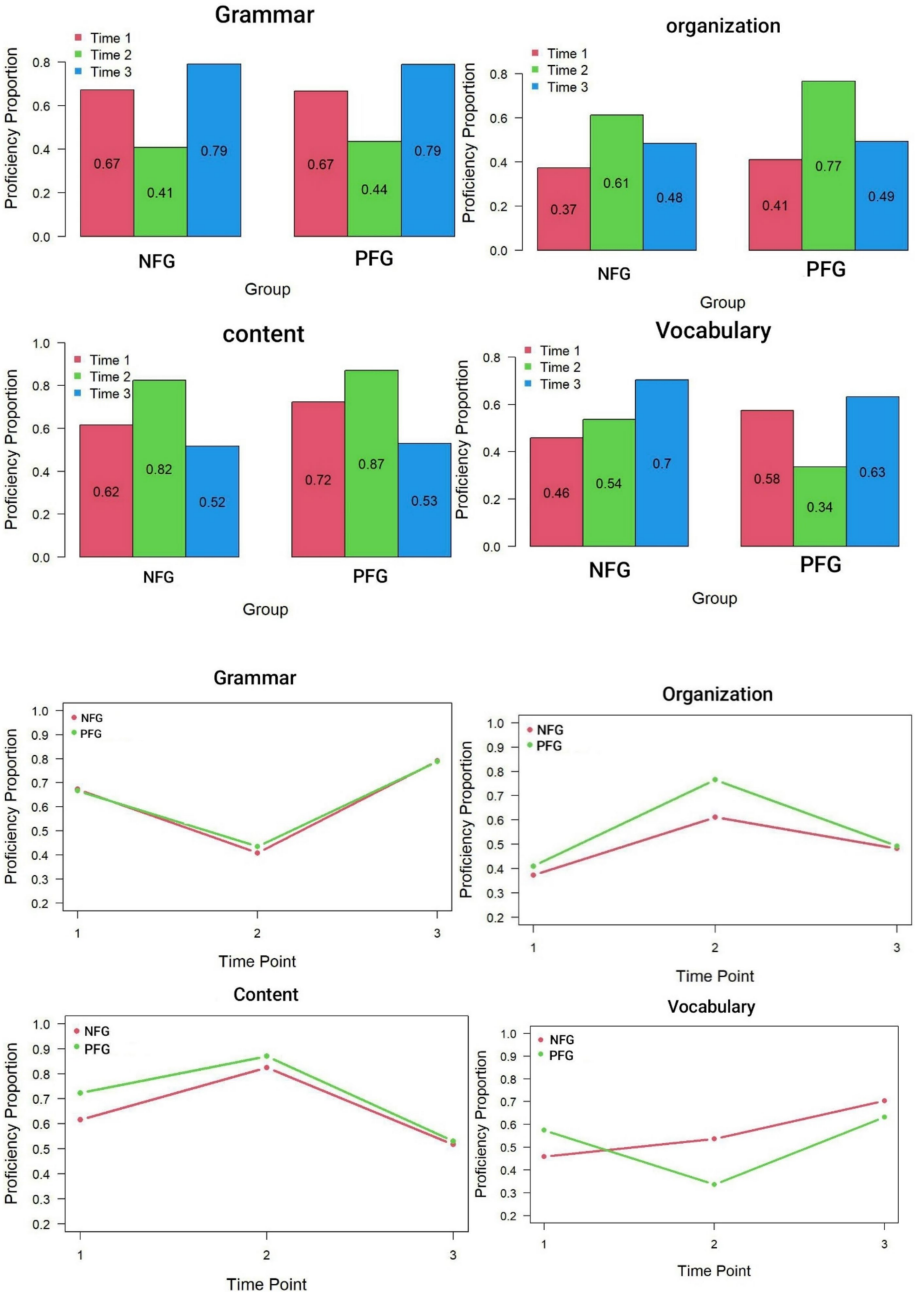
Bar graphs and line graphs displaying growth trajectory of the attributes across groups. NFG, no feedback group; PFG, peer feedback group.

The TDCM generates transition probabilities of each subskill for each individual across the various time points. Due to space constraints, the output has not been included here. The TDCM also generates bar graphs and line graphs for each group separately. Bar graphs and line graphs for the two groups across different attributes and time points are illustrated in Figure 1. Visually inspecting the bar graphs, one notices that the growth pattern in Grammar and Vocabulary were strikingly similar, and the pattern of Organization and Content were intriguingly mirror images of each other. This observation suggests a potential similarity or commonality between these pairs of attributes.

The pattern observed in the bar graphs is mirrored in the line graphs. For Grammar and Vocabulary, the trajectory formed a V-shaped pattern, while for Organization and Content, it resembled an inverted V. Specifically, for Grammar, there was a sharp decline towards the second time point, followed by a steep rise towards the third time point. Along this trajectory, the lines representing the two groups closely paralleled each other, indicating similar mastery levels. Vocabulary displayed a similar pattern, although the control group’s trajectory showed a smooth upward trend from Time 1 to Time 3. Organization and Content depicted the two groups starting with nearly identical proficiency levels, experiencing a sharp rise at Time 2 followed by a steep decline. According to the line graphs, the intervention seemed to have a transient effect on attribute development, with subjects ultimately reaching mastery levels closely resembling their initial proficiency.

## 6 Discussion

The present study aimed to demonstrate the application of a growth DCM in tracking the development of attributes over time, using the TDCM package. To achieve this, writing data from two groups of university students participating in an experimental study—exploring the impact of different types of feedback on their writing development across three distinct time points—were analyzed. A multigroup analysis was conducted, and interpretation of the findings was presented.

Although the primary objective of this paper was to illustrate how the TDCM package can be used to measure growth over time, a substantive discussion of the results may offer valuable insights into how the findings from longitudinal DCMs can be interpreted to study learning. However, before delving into the results, it is important to acknowledge a caveat: the small sample size could potentially compromise the accuracy of the findings. Therefore, the substantive results should be interpreted with caution.

The two-group analysis showed that item parameters varied across time but remained consistent across groups. The findings also indicated that both Vocabulary and Grammar exhibited similar developmental trajectories in the PFG and NFG. Similarly, consistent patterns were observed for Content and Organization. Overall, PFG did not result in a distinct growth trajectory compared to the NFG. This lack of effect can be explained by learners’ beliefs about feedback. In a review study of feedback, Winstone et al. (2017) discovered that the effectiveness of feedback is significantly influenced by individual differences among learners, including their attitudes, beliefs, and goals. Similarly, Carless and Boud (2018) highlighted the crucial role of learners in actively engaging with and utilizing feedback in higher education settings. Research by Yoshida (2008) indicated that many learners harbor negative perceptions of peer feedback, often questioning the accuracy of their peers’ evaluations. Sato (2017) suggested that the effectiveness of peer feedback is influenced by social factors such as distrust in peers’ language skills, discomfort in giving feedback, and embarrassment when being corrected by peers. Although the present study did not measure the subjects’ attitudes towards peer feedback, it is likely that these social dynamics limited the impact of peer feedback on the improvement of various subskills.

The line graphs depicting the growth trajectories of the attributes across timepoints and groups revealed distinct patterns: Grammar and Vocabulary exhibited a V-shaped trajectory, while Organization and Content followed an inverted V pattern. The observed regression or loss of proficiency in Grammar and Vocabulary at Time 2 can be attributed to the participants’ status as sophomore university students taking their first writing course. Before this course, they had already completed Grammar courses I and II, as well as Reading Comprehension Courses I and II, and in preparation for the university entrance examination, they had focused on grammar, vocabulary, and reading comprehension. Consequently, their initial proficiency in Grammar and Vocabulary was relatively high.

However, as the course progressed, they were introduced to Content and Organization. According to *Cognitive Load Theory* (Sweller, 1988), learners have a limited cognitive capacity for processing new information. The introduction of new subskills such as Content and Organization likely increased the cognitive load, causing a temporary decline in their mastery of Grammar and Vocabulary. This cognitive overload at the initial stages of the intervention could explain the regression observed in these areas.

Furthermore, *Limited Attentional Capacity theory* (Kahneman, 1973) supports the notion that individuals have finite attentional resources. Initially, the learners may have struggled to allocate their attention effectively between the newly introduced subskills (Content and Organization) and the previously acquired skills (Grammar and Vocabulary). This imbalance likely led to a decline in Grammar and Vocabulary proficiency as they concentrated more on developing Content and Organization skills.

Proficiency levels increased for Content and Organization subskills at Time 2, suggesting that learners allocated more attention to these less familiar areas. The intervention may have initially provided new insights or techniques, leading to rapid gains in these areas. However, this focus on Content and Organization came at the expense of Grammar and Vocabulary. As learners adapted and practiced over time, they gradually learned to balance their attentional resources more effectively. This reallocation of attention resulted in improved proficiency in Grammar and Vocabulary at Time 3, alongside a decline in Content and Organization proficiency.

This pattern aligns with Cognitive Load Theory, which posits that effective learning requires managing cognitive load to prevent overload. It also aligns with Limited Attentional Capacity theory, indicating that learners needed time to distribute their attentional resources across all subskills more effectively. The results suggest that learners had not yet achieved mastery over all four subskills simultaneously, highlighting the ongoing challenge of balancing cognitive and attentional demands in the process of skill acquisition.

To attain a more comprehensive understanding of the trajectories for all four subskills, it is advisable to collect data from subsequent time points beyond Time Point 3. This trajectory of Content and Organization proficiency mirrors patterns typically observed in experimental designs featuring pre-tests, post-tests, and delayed post-tests. While learning or mastery typically increases on the post-test due to the intervention, retention issues may lead to a decline in performance on the delayed post-test.

The implication of observing V-shaped and inverted V-shaped patterns of development for experimental studies is that those studies which only capture language skill development at two time points, as is often the case in many experimental studies, may present an incomplete and potentially misleading picture. According to Larsen-Freeman (2011), different parts of the language system are acquired at different rates. Development often includes bursts of rapid progress, interspersed with periods of stability or regression, and subsequent phases of continued advancement (Jia and Fuse, 2007). Consider a learner who has been taught how to negate in English. In the early stages of learning, she memorizes negative statements as chunks and performs well on the post-test (Time 2). However, as the learners begin to unpack the formulaic phrases, their performance may decline on the delayed post-test before mastering them fully.

The implications of this study underscore the necessity of adopting longitudinal approaches in assessing language skill development, particularly within the context of writing. While conventional pre-test/post-test designs are prevalent in intervention studies, they may offer only a partial view of skill acquisition. It is crucial to acknowledge that the effects of instructional interventions may not be immediately apparent, as learners require time to integrate newly acquired skills into their linguistic repertoire. Thus, longitudinal studies provide a more comprehensive understanding of skill growth by capturing the trajectory of change over time. By observing learners across multiple time points, researchers can discern between short-term improvements and enduring changes, thereby offering a more accurate assessment of intervention efficacy. This approach ensures an understanding of language skill development and underscores the importance of incorporating temporal dynamics into research methodologies. Additionally, individual differences in learning styles, motivation, and cognitive abilities could also interact with attributes or subskills and account for these contrasting patterns.

Before wrapping up this section, we should note that the finding that Multigroup 3 fitted the data, but Multigroup 4 did not is counterintuitive because, theoretically, a less restrictive model (Multigroup 4) should fit the data at least as well as, if not better than, a more restrictive model (Multigroup 3). Multigroup 4 imposed fewer constraints by allowing item parameters to vary freely across both groups and time points, offering greater flexibility to capture the nuances of the data. In contrast, Multigroup 3 restricted item parameters to be invariant across groups, which limits its ability to account for between-group differences. Generally, greater flexibility in model parameters tends to improve model fit because it accommodates more variability in the data. Hence, it is unexpected that the more constrained Multigroup 3 model fits well while the less constrained Multigroup 4 model does not. The finding can be attributed to several potential reasons. First, while Multigroup 4 allows for complete flexibility in item parameters across groups and time points, this added complexity may lead to overparameterization. Overly complex models are more prone to capturing random noise or idiosyncratic patterns rather than meaningful variance, which can result in poorer fit indices (Marsh et al., 2004; Kline, 2016). Second, the sample size and distribution of responses could play a role. Insufficient data may fail to support the additional parameters estimated in Multigroup 4, potentially causing convergence issues or unstable estimates, which can adversely affect model fit (Bentler, 1990).

## 7 Concluding remarks

The present study encountered several limitations, which future research can address to improve upon its findings. One notable limitation is the relatively small sample size, which may have contributed to the convergence issues observed. DCMs typically require larger sample sizes to ensure stable and reliable results (Ravand and Robitzsch, 2018).

Another limitation, common in performance assessment studies utilizing DCMs (including the present study), is the lack of integration of rater effects into the analysis. This omission could potentially compromise the validity of the results.

Additionally, the checklist used to assess the writings in this study employed a dichotomous scoring approach. Treating a fundamentally non-dichotomous, continuous construct like writing proficiency as dichotomous may undermine the accuracy of student classifications (Tu et al., 2017). As Karelitz (2008) argues, reducing an ability continuum to binary categories of mastery/non-mastery risks obscuring the intermediate stages of development. This approach contradicts the widely accepted view in second language acquisition research that language learning is a gradual and progressive process. To address this issue, future studies could consider rating students on a scale, such as 1 to 5, for each descriptor and applying polytomous CDMs (e.g., Ma, 2019; Ma and de la Torre, 2016) for analysis. This approach would provide a more nuanced understanding of learners’ writing proficiency and align better with second language acquisition research principles.

## Data Availability

The data analyzed in this study is subject to the following licenses/restrictions: the datasets generated during and/or analyzed during the current study are available from the first author on reasonable requests. Requests to access these datasets should be directed to ravand@vru.ac.ir.

## Author’s note

In this paper, “attribute” and “subskill” are used interchangeably to denote the discrete latent variables predicting performance on each item/descriptor.
