# Supplementary material for: A didactic illustration of writing skill growth through a longitudinal diagnostic classification model
Source: Front Psychol. 2025 Jan 15;15:1521808. doi: 10.3389/fpsyg.2024.1521808 (PMC11775902; doi:10.3389/fpsyg.2024.1521808)
Supplement: Supplementary file 1 [file Data_Sheet_1.docx]

**SINGLE GROUP**

*model1 <- tdcm(data, q.matrix, num.time.points = 3)*

#estimates a model with three data points and the item parameters are assumed to be invariant across the time points (Model A in Table 1 in the paper)

*model2 <- tdcm(data, q.matrix, num.time.points = 3,invariance=F)*

# estimates a model with three data points and the item parameters are assumed to be varying across the time points (Model B in Table 1 in the paper)

*anova(model1,model2)*

# compares two models, where one is nested in the other one.

*results1 <- tdcm.summary(model1, num.time.points = 3, transition.option = 3)*

# after model1 has been estimated, the above code summarizes the results. It should be noted the default for the transition.option is 1, if sepecified so, transition probabilities at Time 1 are compared against those of the last time point (here Time 3). transition.option = 2 compares the first time point to every other time point and transition.option = 3 compares successive time points.

*results1$model.fit$Global.Fit.Stats2*

# esitmates absolute fit indexes

*results1$reliability*

#estimates transition reliabilities

*item.parameters <- results1$item.parameters*

*item.parameters*

#generates item parameters. If a model with the assumption of the invariance of item parameters has been estimated, one set of parameters will be produced, otherwise the number of item parameter sets equals the number of time points

*growth <- results1$growth*

*growth*

#gives the average mastery for each subskill/attribute across the time points

*transition.probabilities <- results1$transition.probabilities*

*transition.probabilities*

#estimates the probability of transitioning from one time point to the next averaged across all the examinees

*transition.posteriors <- results1$transition.posteriors*

#estimates the probability of transitioning from one time point to the next for each individual examinee

*tdcm.plot(results1, attribute.names = c("Attribute1","Attribute2","Attribute3","Attribute4"))*

#generates bar graphs and line graphs

**Different Q-matrices with Anchor Item**

When a different test is used at different time points one need to specify the number of Q-matrices and the number of items at each time point.

*mg1 <- tdcm(data , q.matrix, num.time.points = 3,num.q.matrix=3,num.items = c(20,20,20),anchor=c(3,23,42,5,25,45,7,27,47),rule = "GDINA")*

# The provided code estimates a model with three different time points, each with a different test specified by the parameters num.q.matrix=3 and num.items = c(20,20,20). In this setup, for scaling purposes, it's essential to have common items, referred to as anchor items, across the time points. In the code snippet, items 3, 5, and 7 are designated as common anchor items. To ensure compatibility with the TDCM package, the data file should be structured such that the items across all time points follow the same numbering system. For instance, if there are 20 items in each time point, items should be numbered from 1 to 20 for Time Point 1, 21 to 40 for Time Point 2, and so on. The code anchor=c(3,23,43,5,25,45,7,27,47) specifies that Item 3 at Time Point 1 corresponds to Item 23 at Time Point 2, and Item 43 at Time Point 3, and similarly for Items 5 and 7.

**Multigroup**

*mg1 <- mg.tdcm(data, q.matrix, num.time.points = 3, rule = "GDINA", groups = groups, group.invariance = TRUE, item.invariance = TRUE)*

# The code estimates a multigroup TDCM, where item parameters remain invariant across both groups and time points. Since this is the default setting for the multigroup TDCM, the snippet group.invariance = TRUE, item.invariance = TRUE can be omitted.

*mg2 <- mg.tdcm(data, q.matrix, num.time.points = 3, rule = "GDINA", groups = groups, group.invariance = FALSE, item.invariance = TRUE)*

#estimates a multigroup TDCM where item parameters are varying across groups but invariant across the time points. Hence, in such a model, *n* sets of item parameters (where *n* equals the number of groups) will be estimated.

*mg3 <- mg.tdcm(data, q.matrix, num.time.points = 3, rule = "GDINA", groups = groups, group.invariance = TRUE, item.invariance = FALSE)*

# The code estimates a multigroup TDCM where item parameters are invariant across groups but vary across time points. Consequently, for a study with three time points, the code generates three sets of item parameters: one for each time point.

*mg4 <- mg.tdcm(data, q.matrix, num.time.points = 3, rule = "GDINA", groups = groups, group.invariance = FALSE, item.invariance = FALSE)*

#estimate a multigroup TDCM in which item parameter vary across both groups and time points. Consequently, with three time point and two groups the code estimates six sets of item parameters, one for each combination of group and time point.

*tdcm.compare(mg1, mg2)*

*tdcm.compare(mg1, mg3)*

*tdcm.compare(mg1, mg4)*

#the above codes compare the fit of each model against the model which assumes item parameter invariance across groups and time point.

*Resultsmg3 <- mg.tdcm.summary(mg3, num.time.points = 3, transition.option = 3, attribute.names = c("Attribute1", " Attribute2", " Attribute3", " Attribute4"))*

#summarizes the results of the multigroup TDCM

*resultsmg3$item.parameters*

#yields item parameters

*resultsmg3$growth*

# gives the average mastery for each subskill/attribute across the time points separately for each group

*resultsmg3$transition.probabilities*

# estimates the probability of transitioning from one time point to the next averaged separately across all the examinees within each group.

*resultsmg3$transition.posteriors*

#estimates the transition probabilities for each individual

*resultsmg3$reliability*

# The code estimates the transition reliabilities for each group as they transition from one time point to the next.

*tdcm.plot(resultsmg3, attribute.names = c("Attribute1","Attribute2","Attribute3","Attribute4"),group.names = c("Control", "Treatment"))*

# generates bar graphs and line graphs for the two groups
